# Unconventional Transport Routes of Soluble and Membrane Proteins and Their Role in Developmental Biology

**DOI:** 10.3390/ijms18040703

**Published:** 2017-03-25

**Authors:** Andrea Pompa, Francesca De Marchis, Maria Teresa Pallotta, Yoselin Benitez-Alfonso, Alexandra Jones, Kerstin Schipper, Kevin Moreau, Viktor Žárský, Gian Pietro Di Sansebastiano, Michele Bellucci

**Affiliations:** 1 Institute of Biosciences and Bioresources—Research Division of Perugia, National Research Council (CNR), via della Madonna Alta 130, Perugia 06128, Italy; andrea.pompa@ibbr.cnr.it (A.P.); francesca.demarchis@ibbr.cnr.it (F.D.M.); 2 Department of Experimental Medicine, University of Perugia, Perugia 06132, Italy; maria.pallotta@unipg.it; 3 Centre for Plant Science, School of Biology, University of Leeds, Leeds LS2 9JT, UK; Y.Benitez-Alfonso@leeds.ac.uk; 4 School of Life Sciences, University of Warwick, Coventry CV4 7AL, UK; alex.jones@warwick.ac.uk; 5 Institute for Microbiology, Heinrich Heine University Düsseldorf, Düsseldorf 40225, Germany; kerstin.schipper@uni-duesseldorf.de; 6 Clinical Biochemistry, Institute of Metabolic Science, University of Cambridge, Cambridge CB2 1TN, UK; km510@cam.ac.uk; 7 Department of Experimental Plant Biology, Faculty of Science, Charles University, Prague 2 12844, Czech Republic; zarsky@ueb.cas.cz; 8 Institute of Experimental Botany, v.v.i., the Czech Academy of Sciences, Prague 6 16502, Czech Republic; 9 Department of Biological and Environmental Sciences and Technologies (DISTEBA), University of Salento, S.P. 6, Lecce 73100, Italy; gp.disansebastiano@unisalento.it

**Keywords:** protein secretion, intercellular channels, exosomes, autophagy, trafficking mechanisms, leaderless proteins, unconventional secretion

## Abstract

Many proteins and cargoes in eukaryotic cells are secreted through the conventional secretory pathway that brings proteins and membranes from the endoplasmic reticulum to the plasma membrane, passing through various cell compartments, and then the extracellular space. The recent identification of an increasing number of leaderless secreted proteins bypassing the Golgi apparatus unveiled the existence of alternative protein secretion pathways. Moreover, other unconventional routes for secretion of soluble or transmembrane proteins with initial endoplasmic reticulum localization were identified. Furthermore, other proteins normally functioning in conventional membrane traffic or in the biogenesis of unique plant/fungi organelles or in plasmodesmata transport seem to be involved in unconventional secretory pathways. These alternative pathways are functionally related to biotic stress and development, and are becoming more and more important in cell biology studies in yeast, mammalian cells and in plants. The city of Lecce hosted specialists working on mammals, plants and microorganisms for the inaugural meeting on “Unconventional Protein and Membrane Traffic” (UPMT) during 4–7 October 2016. The main aim of the meeting was to include the highest number of topics, summarized in this report, related to the unconventional transport routes of protein and membranes.

## 1. Introduction

The definition of the conventional secretory pathway arose from studies realized during the 1960s and 1970s (reviewed in [1]). It is a eukaryotic metabolic transport pathway that brings proteins harboring a N-terminal signal peptide, which mediates protein translocation in the lumen or the membrane of the endoplasmic reticulum (ER), from the ER to the Golgi apparatus, subsequently to the trans-Golgi network and then to the plasma membrane (PM), where proteins are released into the extracellular space. Multiple rounds of sequential budding and fusion of vesicular carriers mediate protein secretion among compartments [2]. Membrane proteins, for example integral PM proteins, are also delivered to their target membrane through this secretory pathway, referred to as conventional protein secretion by some authors [3]. The conventional secretory pathway includes also the traffic of proteins to the vacuole/lysosome, since these proteins move through the ER and Golgi apparatus before being segregated in the trans-Golgi network from the other two protein destinations; PM and endosomes [1,3]. The role of the conventional secretory pathway in the life of an organism is fundamental because it regulates many physiological processes like growth, defense, hormone release, cell homeostasis, and reproduction among others.

**Figure 1:**
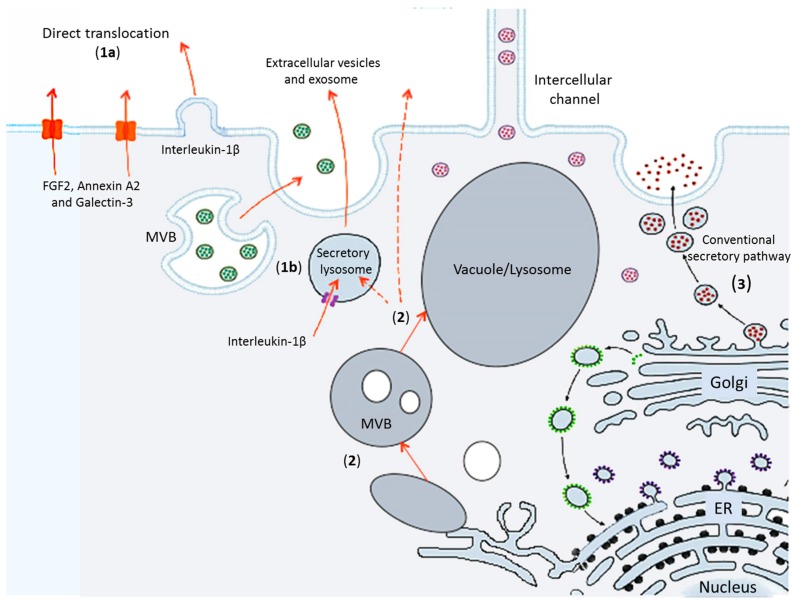
Schematic representation of secretory pathways within the eukaryotic cell. Some of the UPS pathways are indicated (see text for more details): (**1**) Leaderless proteins directly translocated across the PM, by means of non-vesicular (**1a**) and vesicular (**1b**) UPS pathways. Examples are proteins FGF2, Annexin A2, Galectin-3 and Interleukin-1β. (**2**) Soluble or transmembrane proteins with ER localization subsequently transported to the PM, or to the vacuole, or to the extra cellular space (by using or not using secretory lysosomes, dashed lines) bypassing the Golgi apparatus. In addition, the conventional secretory pathway is also indicated (**3**), but it should be considered that some proteins normally functioning in the conventional membrane traffic can have an additional unconventional role. MVB, multivesicular bodies. Modified from [7].

**Figure 2:**
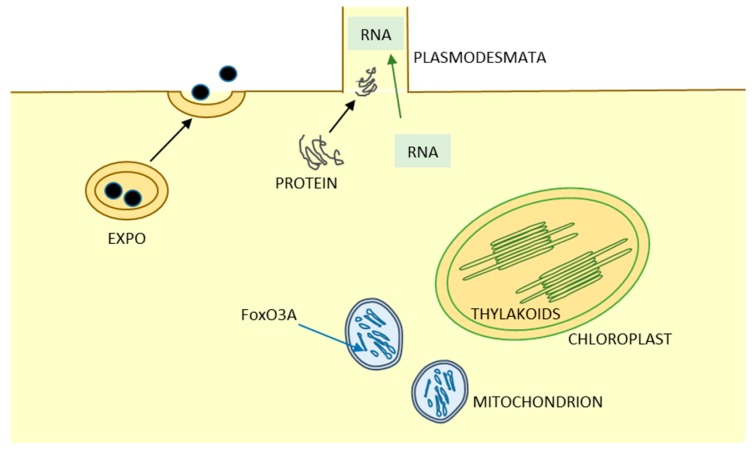
Some examples of unusual or unexplored intra- and intercellular pathways and organelle biogenesis. During the meeting the participants underlined that intra- and intercellular pathways, including organelle biogenesis and plasmodesmata, can represent other types of UPS pathways (see Section 4 and Section 6 in the text). An example of vesicular UPS mediated by a plant-specific double-membrane-bound compartment is the contentious exocyst-positive organelle (EXPO), which biogenesis seems to be mediated by a UPS pathway. Moreover, in eukaryotes RNA and proteins transported by intercellular channels regulate different processes in relation to trafficking mechanisms. In mammalian cells, another example is the unconventional transport of FoxO3A, a transcription factor working in normal conditions on genomic DNA in the nucleus, to mitochondria, while in plants chloroplast vesicle transport and thylakoid biogenesis is under investigation to identify protein factors involved in the formation of this unusual membrane system.

Recently, the identification of an increasing number of secreted signal peptide-lacking proteins, also called leaderless secretory proteins, revealed the existence of unconventional protein secretion (UPS) pathways where these proteins bypass intermediate compartments involved in secretion or exocytosis, such as the Golgi apparatus [4]. Current studies are increasing the number of proteins known to traffic through the UPS pathways which can be broadly categorized as following: (i) leaderless proteins directly secreted and translocated across the PM, by means of vesicular and non-vesicular UPS pathways; (ii) soluble or transmembrane proteins with ER localization subsequently transported to the PM, or to the vacuole, or to the extra cellular space bypassing the Golgi apparatus; (iii) proteins normally functioning in the conventional membrane traffic with an additional unconventional role; and (iv) proteins involved in unusual or unexplored intra- and intercellular pathways and organelle biogenesis (Figure 1). Some scientists underlined that also intercellular channels represent a route for the transport of proteins and other macromolecules, largely independent of conventional secretory pathway, and therefore can represent another type of UPS pathway (Figure 2). Because of the large number of UPS pathways, Devis and colleagues stated that “one is tempted to speculate that what is termed unconventional protein secretion may not be that unconventional after all” [5]. It is therefore clear that UPS is becoming more and more important in cell biology studies, which were initially carried out by yeast and mammalian cell biologists [6,7], but, recently, insights from plant biologists have contributed to this topic [5]. Trafficking of proteins and membranes with an unconventional role is related to human pathologies as well as to plant biotic stress and development. Thus, combining expertise and research efforts from different fields to develop an open comparative approach to tackle these subjects is timely. The meeting on “Unconventional Protein and Membrane Traffic” (UPMT) held in Lecce during 4–7 October 2016 tried to address this topic and generate an open discussion among specialists working on mammals, plants and microorganisms.

During the four days, the meeting was characterized by presentations grouped in seven sessions to cover topics related to the definition of UPS in a broad sense, and these presentations are summarized in this report organized into five sections (from Section 2, Section 3, Section 4, Section 5 to Section 6). The speakers gave very interesting talks stimulating interactive discussions that continued during the poster session. To examine in depth the subject of UPS, we recommend other review articles published here in the same Special Issue of International Journal of Molecular Sciences, in addition to the review articles already cited in this Introduction.

## 2. Routes for Unconventional Secretion of Leaderless Proteins to the Extra Cellular Space

Most eukaryotic leaderless cytoplasmic proteins reach the cell surface by UPS. Such proteins include members of the Annexin family, cytoskeletal proteins, Heat Shock Proteins, members of the interleukin family, fibroblast growth factors, and others (see review [8] for references). When attached to the outer leaflet of the PM, some of these proteins can then be cleaved by extracellular proteases and released into the extracellular environment. In the extracellular space, these macromolecules have been shown to have crucial roles in normal physiology as well as in human diseases such as cancer, infection, and metabolic and autoimmune disorders [9,10,11,12,13,14,15]. In plants, the situation is different because there are many putative leaderless secretory proteins but very few of them have been experimentally demonstrated to localize to the apoplast and their physiological role determined [4]. The only exception now is Helja, a mannose-specific jacalin-related lectin in sunflower seedlings [16].

UPS can occur by different modes, some proteins can directly translocate across the PM but others could become membrane encased and then secreted through extracellular vesicles, such as exosomes or ectosomes, and/or lysosomes [8,17]. Intraluminal vesicles, generated within multivesicular bodies, are secreted in the extracellular milieu when multivesicular bodies fuse with the PM and are therefore termed exosomes. In contrast to exosomes, ectosomes are directly formed at the PM. Extracellular vesicles can be classified in various subtypes according to their size, intracellular origin, presence of specific markers, and timing of release [18,19]. Jacopo Meldolesi (San Raffaele Institute, Milano, Italy) introduced this topic in the meeting, describing EV generation, release and fusion with target cells, and illustrating his vision gained during his long research career in the field [20]. However, the same protein can be also secreted in different ways. For example, different human cell types and culture conditions support secretion of Interleukin-1β. Anna Rubartelli (IRCCS AOU San Martino—IST, Genova, Italy) reviewed the multiple ways proposed for Interleukin-1β secretion, including vesicle exocytosis or release, autophagy, leakage through pores in the PM in a process involving Gasdermin-D and pyroptosis, with a special focus on the role of redox response and on different inflammatory responses [21,22,23].

### 2.1. Pore Formation at the PM

**Figure 3:**
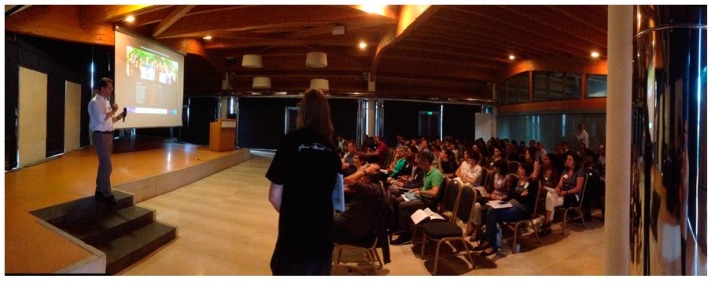
A picture showing Walter Nickel during his talk.

One of the direct UPS translocation mechanisms required the formation of a pore at the PM, as summarized by the group of Walter Nickel (Heidelberg University, Heidelberg, Germany). They discussed unpublished data on the direct transport of the cytoplasmic protein fibroblast growth factor 2 across the PM. This transport, as reported by Nickel (Figure 3), requires fibroblast growth factor 2 recruitment to the PM along with its Tec kinase dependent tyrosine phosphorylation, phosphoinositide PI(4,5)P2-dependent oligomerization and membrane pore formation, and extracellular trapping by cell surface heparin sulfate proteoglycans mediating fibroblast growth factor 2 translocation into the extracellular space [24,25,26]. An elegant approach to visualize the translocation of fibroblast growth factor 2-GFP across the PM using live cells imaging was also presented by Eleni Dimou (Nickel group) based on the utilization of anti-GFP nanobodies that should considerably improve the understanding of the spatio-temporal coordination and the molecular mechanism of fibroblast growth factor 2 secretion. The importance of fibroblast growth factor 2 oligomerization during the translocation process was further developed in a separate talk by Sebastian Unger (Nickel group) showing the requirement of two surface cysteines that are uniquely present in fibroblast growth factor 2, but not in other fibroblast growth factor family members carrying signal peptides. Cell surface proteoglycans seems to be important for secretion of the cytoskeletal protein tau, as described also for fibroblast growth factor 2. Taxiarchis Katsinelos, from Thomas Jahn’s group (DKFZ Heidelberg University, Heidelberg, Germany), talked about his work on the secretion and the trans-cellular propagation of Alzheimer’s disease associated tau protein [27]. A major hallmark of Alzheimer’s disease is the progressive accumulation of the microtubule-associated tau protein. However, the mechanisms for tau secretion are poorly understood. Using an inducible cell culture system to analyze tau secretion, they could correlate the phosphorylation status of tau with its aggregation propensity and secretion efficiency. The secreted tau was mainly found as free soluble protein and was not vesicle associated.

Another likely example of direct translocation by pore formation was showed by Kevin Moreau (University of Cambridge, Cambridge, UK) regarding the transport to the cell surface of Annexin A2 and Galectin-3. Cell surface Annexin A2 acts as a plasminogen receptor able to stimulate fibrinolysis and cell migration through the extracellular matrix proteolysis while cell surface Galectin-3 serves as a cell-to-cell and cell-to-matrix adhesion/interaction molecule [8]. He presented data identifying key regulators of Galectin-3 localization to the cell surface using a genome-wide screen based on CRISPR-Cas9 technology. Moreover, using a combination of biochemical, biophysical and cell biological approaches, he showed that the translocation of Annexin A2 across membranes requires phospholipid binding and lipid reorganization. Interestingly, Pablo Pelegrin (IMIB-Arrixaca, Murcia, Spain) identified the P2X7 receptor in regulating Annexins secretion during macrophage polarization [28] and discussed this in the context of Annexins translocation across membranes. The purinergic receptor P2X7R once activated promotes the assembly of the NLRP3 inflammasome and the unconventional release of the pro-inflammatory cytokines Interleukin-1β and Interleukin-18 from macrophages [28]. In this study, he characterized P2X7R secretome and found the release of novel cell-related proteins.

Members of the interleukin family are a case study for this kind of UPS. Interleukin-1β is an essential mediator of inflammation after infection and injury. This cytokine is produced as an inactive precursor, which must be cleaved to its active form via a molecular complex called the inflammasome. Interleukin-1β is released from the cell via an unknown process. The group of David Brough (with the presentations of Jack Rivers-Auty, Michael Daniels, and Catherine Diamond, University of Manchester, Manchester, UK) discussed its recent publication about the release of Interleukin-1β via a mechanism that requires membrane permeabilization [29]. The mechanism of membrane permeabilization required for Interleukin-1β release seems to be distinct from the formation of membrane pores required for other types of unconventional protein secretion that do not result in cell death but still needs further characterization. Michael Daniels also talked about the unusual nuclear localization of Interleukin-1α due to a nuclear localization sequence at the N-terminus that is important for its function. He further discussed this observation in the context of evolution.

### 2.2. Different Types of Secretory Vesicles for Leaderless Proteins Translocation

Interleukin-1β can be also be secreted by a vesicular mean of transport. In human monocytes, Interleukin-1β secretion is mediated by secretory lysosomes, induced by exogenous ATP, once more underlining the complex crosstalk among different secretory mechanisms [22]. However, other vesicular pathways seem to be involved in UPS. Claudia Verderio (Institute of Neuroscience, CNR, Milano, Italy) stressed the importance of extracellular vesicles as carriers of signals. Microglia-derived extracellular vesicles store inflammatory signals and propagate an inflammatory response to other glial cells, enhancing spontaneous and evoked excitatory transmission [30,31,32]. The meeting was an occasion to discuss the molecular mechanism supporting propagation of synaptic dysfunctions among connected neurons through extracellular vesicles. A deeper knowledge on their behavior can have a tremendous impact on medical research because microglia-derived extracellular vesicles concentration in the cerebrospinal fluid correlates with classical markers of neurodegeneration and Alzheimer’s disease [33,34]. However, the formation of multivesicular bodies releasing exosomes is far from being fully elucidated.

Autophagic vesicles could be also used for unconventional protein secretion from the cell. For example, William Jackson (University of Maryland, Baltimore, MD, USA) described how Poliovirus 1, a Picornavirus responsible for human disease, uses autophagic vesicles to promote virus replication and exit from the cell [35]. In particular, the maturation and acidification of autophagic vesicles into amphisomes by fusion with the lysosome benefits Poliovirus 1 by promoting maturation of the viral particle. Jackson demonstrated that functionally eliminating specific proteins of a complex that mediates vesicle fusion (SNARE complex), Poliovirus 1 and other picornaviruses might enhance unconventional autophagic secretion to promote viral exit from cells.

A novel compartment, formed by a tubulovesicular structure surrounded by a cup-shaped membrane, named compartment for unconventional protein secretion (CUPS), is induced in yeast by nutrient starvation triggering secretion of the signal sequence-lacking Acb1 protein. The biogenesis of the compartment for unconventional protein secretion requires several proteins, including an ER exit site/Golgi resident protein, Grh1. Amy Curwin (Centre for Genomic Regulation, Barcelona, Spain) from the Malhotra lab reported that the major endosomal sorting complex required for transport-III subunit Snf7 localizes transiently to this novel compartment, and promotes direct engulfment of preexisting Grh1 containing vesicles and tubules into a saccule to generate a mature compartment for unconventional protein secretion containing Acb1 [36,37]. The Malhotra group suggest that this novel multivesicular compartment is the stable secretory form releasing Acb1-containing exosome-like vesicles into the extracellular space where they lyse to release Acb1.

### 2.3. Other Examples of Unconventional Leaderless Proteins Secretion

Kerstin Schipper (Heinrich Heine University, Düsseldorf, Germany) presented new data on the chitinase Cts1 from the fungus *Ustilago maydis* that lacks a classical N-terminal secretion signal but is secreted in the fragmentation zone between mother and daughter cells during cytokinesis [38]. Her team has established an ingenious reporter system to test if secreted proteins pass through the ER [39]. The bacterial enzyme β-glucuronidase (GUS) is widely used as a reporter of gene activity as it can catalyze the conversion of the colorless substrate 5-bromo-4-chloro-3-indolyl glucuronide to a blue product. GUS contains a eukaryotic *N*-glycosylation signal, but—crucially—glycosylation largely inactivates the enzyme. Stock et al. realized that this feature could be harnessed to prove that a secreted protein fused to GUS does indeed bypass the ER [40]. Schipper discussed the first results of their genetic screen to identify proteins essential to the unconventional secretion of Cts1, before making a passionate case for the development of *U. maydis* for protein production. Indeed, inappropriate *N*-glycosylation can be a problem in the heterologous production of pharmaceutical proteins. Cts1 can act as a carrier to avoid unwanted ER processing whilst retaining the advantages of protein secretion for facilitated downstream processing. Another leaderless mammalian protein, indoleamine 2,3-dioxygenase 1, seems to be secreted by UPS, even if the exact unconventional mechanism is still unknown. Maria Teresa Pallotta (University of Perugia, Perugia, Italy) presented unpublished data regarding this protein, an enzyme that exerts regulatory functions in autoimmune and inflammatory settings [41]. She demonstrated that a specific extracellular milieu can promote different subcellular localizations and also extracellular secretion of the indoleamine 2,3-dioxygenase 1 enzyme.

It is clear from the above paragraphs that the UPS pathways of leaderless proteins to the extra cellular space are important for the crucial roles of these proteins in normal physiology as well as in human diseases, but also in innate immune response. The majority of examples come from mammalian and yeast biology; therefore, to further characterize these pathways, more research efforts are needed in other disciplines like for example plant biology.

## 3. Routes for Unconventional Secretion of Soluble or Transmembrane Proteins with ER Localization Subsequently Transported to the PM, to the Vacuole, or to the Extra Cellular Space

UPS can take also place by a transportation mode in which both soluble and membrane bound proteins initially enter the conventional secretory pathway whether through ER translocation as they possess ER-targeting signals or through ER association because they are associated with the cytosolic side of ER membranes. Then, these proteins could be subsequently transported to the cell surface or to the vacuole bypassing the Golgi apparatus. There are some examples of animal proteins transported through this kind of pathway [6], but much more examples exist in plants of soluble or membrane proteins, or proteins aggregated into specialized ER vesicles, which are transported from the ER to the vacuole bypassing the Golgi apparatus (reviewed in [42]). Moreover, recent data suggest that newly formed plant lytic vacuoles arise directly from the ER, even though the Golgi apparatus contributes during vacuole formation with the delivery of soluble cargoes and proteins for the tonoplast, the vacuolar membrane [43]. Autophagy is also one possible source for double membrane compartments bypassing the Golgi apparatus in transporting specific cargos directly from ER to the lysosome/vacuole or to the extracellular space/cell wall. It is becoming more and more apparent that autophagy-related endomembrane transport processes are an essential part of basal cellular machinery, but the mode of unconventional secretion that is dependent on autophagy is less clear in mechanistic terms, as well as its intricate interrelations with other processes (including late endosome/multivesicular bodies biogenesis).

### 3.1. Involvement of Autophagy in Unconventional Secretion

Mature autophagosomes, the autophagy organelle, are double-membrane vesicles decorated with the autophagy-related (ATG) protein ATG8 on both surfaces. Autophagosomes, moving along the cytoskeletal network, are transported to the tonoplast with which they fuse to deliver autophagic bodies for enzymatic recycling, or to the PM for secretion. These autophagy pathways can be also used in the cell for other purposes. For example, Alexandra Boeske, from Dieter Willbold’s lab (Heinrich Heine University, Düsseldorf, Germany), discussed the function of ATG8 homologs GABARAP proteins in HIV-1 Nef protein pathogenesis and its unconventional secretion as a follow-up inquiry into previously identified HIV-1 Nef interactors. To get the signal peptide lacking HIV-1 Nef protein out of the cell it needs at least one of GABARAP paralogs to be active, forming a complex with HIV-1 Nef in perinuclear region from where it is secreted to the extracellular space via PM localized patches. Interestingly, also GABARAP non-binding mutant HIV-1 Nef was detected within extracellular vesicles indicating a parallel mechanism of HIV-1 Nef GABARAP dependent export, however not dependent on the direct GABARP–HIV-1 Nef interaction. Interestingly, it was recently shown that, in transfected cells, a proportion of Nef localized to ER membranes where Nef physically interacted with the ER chaperone calnexin [44].

### 3.2. Direct Delivery from the ER to the Vacuole

**Figure 4:**
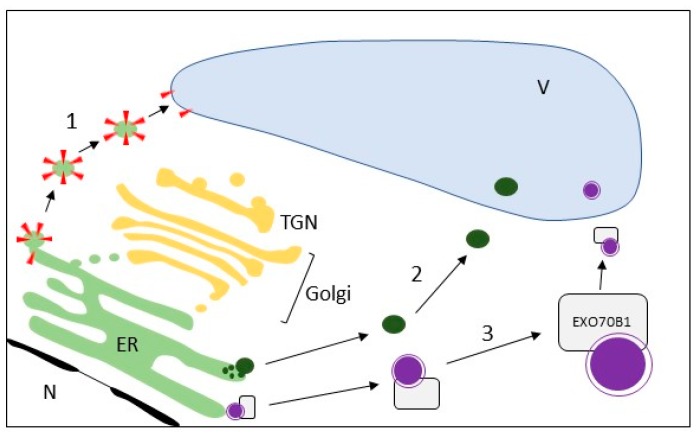
Examples of direct ER to vacuole (V) import routes bypassing the Golgi apparatus and the Trans Golgi Network (TGN) in plant cells. The scarcity of proteins with Golgi-modified glycans (red triangles) on the tonoplast may indicate that the major route (**1**) to the tonoplast bypasses the Golgi apparatus. Moreover, in seed endosperm, some storage proteins are directly transported to the protein storage vacuoles (**2**). Recently, it has been shown that a specific plant exocyst subcomplex, containing EXO70B1, participates in the direct ER to vacuole import of anthocyanins (**3**) which are synthesized by a multienzyme complex loosely associated to the ER.

A new perspective about direct ER to vacuole import (Figure 4) was opened by Viktor Žárský (Charles University and Institute of Experimental Botany ASCR, Prague, Czech Republic). He focused on the observation that a specific plant exocyst subcomplex, containing EXO70B1 [45], participates in the direct ER to vacuole import of anthocyanins. Similar pathway seems to be exploited by some tonoplast proteins. The quantity of proteins with Golgi-modified glycans on the tonoplast is very limited. Animal lysosomes share many characteristics with vacuoles, but the percentage of *N*-glycoproteome of the rat lysosomal and plasma membranes is much higher than that of the plant tonoplast, and is very similar to that of the *Arabidopsis thaliana* PM [46]. Emanuela Pedrazzini (Institute of Agricultural Biology and Biotechnology, CNR, Milano, Italy) suggested that this scarcity of glycoproteins may indicate that the major route to the tonoplast bypasses the Golgi apparatus and, indeed, and increasing number of publications support this hypothesis [46,47,48,49]. The use of a plant experimental system to investigate the UPS mechanisms may also benefit research in the animal field because in animal cells traffic that bypasses the Golgi is evident only during ER stress and autophagy induction [50]. For example, many investigations on lysosome/vacuole traffic started from the study of sorting receptors. Despite the differences in binding mechanisms of cargo to receptors, the signals involved are very similar in human, yeast and plants. These trafficking signals are often located in the C-terminal tails of sorting receptors with dileucine and tyrosine based motifs. Carine de Marcos Lousa (Beckett University, Leeds, UK) described the identification of a specific plant vacuolar sorting receptors (VSR) isoform that follows an alternative route to the vacuole [51]. Interestingly, understanding the possible variability in lysosome/vacuole traffic can shed light on lysosomal sorting diseases, a group of metabolic disorders resulting from lysosomal dysfunction caused by deficiencies in enzyme activity or by trafficking defects. Another interesting experimental system to study protein traffic is the endomembrane system of plant endosperm tissue. Several different storage compartments complicate protein traffic and several different routes were described depending on cell type, developmental stage and environment. Seed storage proteins reach their final destination by two main routes: they travel through ER and Golgi to protein storage vacuoles or accumulate in ER-derived protein bodies. The first route involves post-Golgi multivesicular bodies, but some storage proteins are directly transported to the protein storage vacuoles bypassing the Golgi. It was shown by Verena Ibl (University of Natural Resources and Life Sciences, Vienna, Austria) that the *Hordeum vulgare* Vacuolar Protein Sorting 24 (HvVPS24) has a putative protein sorting role in seed storage protein trafficking in barley endosperm [52]. HvVPS24 is a component of endosomal sorting complex required for transport-III, a large subcomplex essential for biogenesis of MVBs and responsible for protein sorting to vacuoles and to the cell surface.

### 3.3. Exosome Release

Studies on plant-pathogens interactions are producing very interesting results on UPS occurrence in plants mediated by exosome release. Many filamentous pathogens enter plant cells by penetrating the host cell wall. Then they initiate intracellular feeding structures, called haustoria, by invagination of the plant’s PM. Haustorial penetration generates structural and biochemical cascade responses in the plant cells, as a part of the plant innate immune system, which leads to the formation of plants defense structures. During the resistance response a dome-shaped cell wall apposition, named “papilla”, is formed in the outer cell wall of epidermal cells at the site of penetration, which successively evolves to encase the entire haustorial body. Exosome-mediated secretion contributes to papilla formation and multivesicular bodies are often abundant in the plant cytoplasm around papilla [53,54]. Mads Eggert Nielsen (University of Copenhagen, Copenhagen, Denmark) reported that the mere activation of the Rab5 GTPases from the conserved GEF VPS9a by GTP binding is required for pre- and post-invasive immunity against both a non-adapted and, unexpectedly, an adapted powdery mildew fungus in *A. thaliana*. In post-invasive immunity, moreover, VPS9a organizes and mediates cellular endomembrane trafficking promoting the correct delivery of membrane material, likely also from the ER membrane, to the encasement structure.

In both animals and plants, increasing numbers of possible cargoes for the pathways discussed in this section have been discovered. These are often related to the defense against pathogens and, in plants, to transport of seed storage proteins in vacuoles. In all reports unpublished data were in the center of presentations and indicated increasing awareness of cell biology community both working on animals as well as on plant models to the central importance of the endomembrane system and autophagy related processes not only in cytoplasm/proteins degradation, but especially in the formation of endomembrane containers involved also in the UPS.

## 4. Intercellular Channels

Intercellular channels represent a pathway for the transport of proteins, RNA and other macromolecules, largely independent of conventional secretory pathway. These have been identified in both plants and animals where they function in development, response to abiotic stress conditions and diseases. In plants, intercellular channels known as plasmodesmata are inserted in cell wall domains enriched in the polysaccharide callose providing membrane and cytoplasmic continuity for symplastic molecular transport [55,56]. An appressed ER structure (named the desmotubule) traverses the channels but these ER connections do not appear fully functional in intercellular transport. Symplastic communication is restricted by callose accumulation and this pathway plays a role in meristem development, lateral organ formation, bud dormancy, vascular transport and in regulating the spreading of viruses and other pathogens. In animals, intercellular channels named tunneling nanotubes are the main route for long distance macromolecular transport in vitro and in developing embryos. Tunneling nanotubes are long and thin (50–200 nm) membranous protrusions rich in F-actin that appear to transfer cellular components over long distances [57]. As plasmodesmata, tunneling nanotubes play a key role in pathogenesis, enabling movement between cells of viruses, bacteria, and of infectious prion and prion-like proteins.

An entire focus session, chaired by Yoselin Benitez-Alfonso (University of Leeds, Leeds, UK), was dedicated to this topic during the meeting. Emmanuelle Bayer (University of Bordeaux/CNRS, Bordeaux, France) focused on plasmodesmata specialized membrane organization. Electron tomography micrographs revealed details on plasmodesmata ultrastructure as never seen before. Their work shows that within the pores, ER-PM junctions undergo substantial remodeling during cell differentiation and tissue growth that vary from direct membrane contact to intermembrane gap of about 10 nm spanned by spokes. They showed that in newly divided cells, plasmodesmata displayed almost non-existent space between the desmotubule and the plasmodesmata suggesting the absence of cytoplasmic sleeve. Intriguingly, transport of macromolecules was still effective across this type of connections suggesting that there is no simple correlation between ER-PM spacing and the extent of cell-to-cell connectivity. Their work questioned the function of membrane contacts within plasmodesmata.

Chris Hawes (Oxford Brook University, Oxford, UK) presented data on the role of reticulons in cell plates and plasmodesmata formation, suggesting that specific members of the reticulon family (RTN3 and RTN6) target mature plasmodesmata and developing cell plate [58]. Studies on BY2 cells indicated that RTN3 and RTN6 are likely involved in the generation of desmotubules during primary plasmodesmata formation, consistent with overexpression phenotypes showing their capacity to convert ER sheet into tubules. Using RTN for proteomic studies by co-immunoprecipitation, other ER-desmotubule proteins expressed at ER-PM contact sites were identified raising questions on the role of these membrane anchors in cell and plant development [59].

The role of the ER-actin transport network in virus movement through plasmodesmata was presented by Manfred Heinlein (Institut de Biologie Moléculaire des Plantes, CNRS, Strasbourg, France). Tobamoviruses, such as the Tobacco mosaic virus, are transported in the form of viral replication complexes that target plasmodesmata by means of its movement protein [60]. The formation, and intracellular and intercellular trafficking of these complexes relies on the cortical microtubule and ER-actin networks. The formation and anchorage of viral replication complexes occurs at specific cortical platforms where microtubules intersect with the ER membrane (cortical microtubule-associated ER sites) and provide access to microtubule- and actin-motor mediated trafficking required for the recruitment of membranes and host factors. In newly infected cells, some early virus replication complexes detach from their cortical microtubule-associated ER sites and target plasmodesmata, whereas other viral replication complexes remain at their attachment sites and grow to become virus factories that amplify the virus. The Heinlein Lab recently showed that the formation and trafficking of the viral replication complexes and the spread of infection between cells depend on specific myosin motors [61]. Whereas class XI myosins function in viral replication complexes formation and trafficking along the ER membrane between cortical microtubule-associated ER sites towards plasmodesmata, class VIII support a specific step in viral replication complexes transport from the ER into the plasmodesmata. In the absence of class VIII myosin activity the movement protein accumulates in the PM, thus implying a role of the PM during virus movement. The cortical microtubule-associated ER sites are associated with stable ER suggesting their potential relationship to ER-PM contact sites. Moreover, the cortical microtubule-associated ER sites -associated viral replication complexes have a role in dsRNA production, which is known to trigger antiviral RNA silencing and also acts as an elicitor of Pattern-Triggered Immunity [62]. Thus, cortical microtubule-associated ER sites are proposed to represent important subcellular sites of combat between the replicating virus and its host.

Yka Helariutta (Sainsbury Lab University of Cambridge, Cambridge, UK) presented new results on his research on symplastic interfaces and their role in phloem transport. Sieve pores connect the enucleated sieve elements and together with plasmodesmata connections in companion cells they form the route for vascular phloem communication. The work in Helariutta lab carefully dissects the mechanisms underlying the formation of sieve pores and the regulation of plasmodesmata aiming to identify opportunities to engineer long distance signaling. Characterization of proteins, such as the putative choline transporter CHER1 [63], involved in the transition from plasmodesmata to mature sieve pores is combined with genetic analysis of plasmodesmata regulation. Identification of mutants activated in callose biosynthesis (via callose synthase 3, CALS3) indicates the importance of this pathway not only for plasmodesmata but also in vascular transport [64]. Callose accumulation in sieve elements, inhibit periclinal divisions in the vascular tissue as restricts the movement of transcription factors (such as DOF2) that promote procambial cell division. Research on callose roles in modifying cell walls was also presented.

Yoselin Benitez-Alfonso elaborated on plasmodesmata role in development and disease response and presented new evidence that point to plasmodesmata as regulator of beneficial symbiotic interactions. Using proteins involved in the degradation of callose, symplastic transport between epidermal and cortical tissues was enhanced in the model legume *Medicago truncatula*. As a result, root infection and nodulation, after inoculation with the symbiotic nitrogen-fixing bacteria rhizobia, were significantly improved. Her research indicates that callose deposition at plasmodesmata cell walls is significantly downregulated soon after inoculation, concomitant with enhanced symplastic communication. Inducing callose degradation prior inoculation facilitates the establishment of the symbiotic interaction. The identity of the symplastic factors involved and how they regulate root development and response to pathogenic and non-pathogenic microbes are still unknown.

Chiara Zurzolo (Institut Pasteur, Paris, France) talked about the formation, regulation and function of very different type of intercellular channels: tunneling nanotubes in human cells. Different from plasmodesmata in plants, tunneling nanotubes can connect very distant cells but they still transport big macromolecular proteins including GFP. Similarities with filopodia structures, precursors for dendritic spines in neurons [65], suggest a common origin, similar regulatory mechanisms and function but is this supported? She showed that, although both tunneling nanotubes and filopodia require actin for their formation, different remodeling complexes are involved. Specifically, the CDC42/IRSp53/VASP actin regulatory network which promotes filopodia, inhibit tunneling nanotubes function and, conversely, the actin regulatory protein epidermal growth factor receptor pathway 8 (Eps8) which restricts filopodia extension, increases tunneling nanotubes formation. Fluorescent-tagged versions of these proteins were ectopically expressed in neuronal cells and their effects on tunneling nanotubes number and function in vesicle transfer from a “donor” to an “acceptor” population of cells were addressed. Finally, she showed data demonstrating a role for tunneling nanotubes in the intercellular spreading of prion-like aggregated proteins α-synuclein and tau respectively involved in the pathogenesis of Parkinson’s and Alzheimer’s diseases [66,67].

In summary, talks on this focus session contributed to the understanding of the processes underlying the formation, regulation and function of intercellular channels in plants and animals. Moreover, a platform for communication between researchers in tunneling nanotubes and plasmodesmata was established aiming to create common knowledge on these intriguing structures and their roles in UPS pathways.

## 5. Unconventional Role of Proteins Normally Functioning in Conventional Protein and Membrane Traffic

During the meeting, it became apparent that proteins involved in conventional protein and membrane secretion can also play additional unconventional roles, and it was reported in several talks that uncharacterized processes, from exocyst tethering complex formation to the role of interfering SNARE or that of the RAB proteins, can lead to the formation of membranous structures involved in UPS. In the following subsections, are all the different cases belonging to Section 5 discussed in the four-day meeting in Lecce.

### 5.1. Exocyst

The exocyst tethering complex is very recently and surprisingly implicated, not only in exocytosis, but also autophagy in both animals and plants [45,46,47,48,49,50,51,52,53,54,55,56,57,58,59,60,61,62,63,64,65,66,67,68]. Previous indication of exocyst engagement in the autophagy initiation in animals and multiplicity of EXO70 paralogs in land plants both imply a possible role for the exocyst complex as a coordinator contributing to the endomembrane dynamics.

Indeed, Tamara Pečenková (Charles University and Institute of Experimental Botany ASCR, Prague, Czech Republic) discussed the possible role of the plant exocyst complex on transport in defense against pathogens attacks. In plants, the exocyst tethering complex has been shown to participate in numerous events as cell wall differentiation and maturation, cell plate formation, plasma membrane recycling, autophagy and plant defense [45,69]. This great versatility is probably because single copy genes encode just two of the eight subunits of the exocyst complex in *A. thaliana*, whereas there are two or more genes for all the other exocyst components and up to 23 gene members for the EXO70 subunit. Therefore, different exocyst complexes could correspond to the great diversification of endomembrane structure and function in plants. The work of Pečenková and colleagues is focalized on the EXO70B1 and B2, which are highly homologous. Both subunits are involved in defense against pathogens and the EXO70B1 is also implicated in autophagy-related transport to vacuole. Based on preliminary results from microscopic observations they concluded that the role for EXO70Bs could be to support the unconventional cargo transportation and secretion. Rosana Sanchez-Lopez (Universidad Nacional Autonoma de Mexico, Mexico City, Mexico) provided another example of the importance of endomembrane dynamics in biotic interactions by documenting the importance of GNOM related ARF guanine-nucleotide exchange factor (GEF) in nodulation induced in *Vicia faba* by Rhizobacteria.

It would be interesting to test the role of each single component of the exocyst complex with the suite of chemical inhibitors of membrane trafficking discussed by Glenn Hicks (University of California, Riverside, CA, USA). Chemical genomics—the use of small molecule inhibitors combined with genetic screening or reporter systems—is well-established as a tool for investigating endomembrane trafficking in plants. This is particularly true for *A. thaliana*, where a wide variety of reporter lines and characterized cargo proteins are available [70]. He presented new data on endosidin 2 demonstrating that this inhibitor targets a component of the exocyst complex, EXO70A1, to inhibit exocytosis [71].

### 5.2. SNAP29

To explain homo- and heterotypic fusions among large compartments, it will probably be necessary to investigate in more detail the unconventional function of important regulatory proteins such as the soluble NSF attachment protein receptor (SNARE) proteins. These tail anchored proteins are essential for the specificity of vesicular targeting but can also contribute to the definition of membrane identity by assuming alternative roles [72]. An update was presented by Thomas Vaccari (IFOM, Milano, Italy) about the Drosophila Snap29 protein, homolog of the human protein SNAP29, a SNARE protein that localizes to multiple trafficking compartments and is normally required for protein trafficking and for proper GA morphology. It was reported that developing tissue lacking Snap29 accumulates large amounts of autophagosomes, thus highlighting a major role of Snap29 in autophagy and secretion [73]. It was also shown that Snap29 plays a conserved role in formation of the kinetochore for the anchoring of mitotic chromosomes to spindle microtubules [74].

### 5.3. Interfering SNAREs

Another example of an unconventional role for a conventional trafficking protein was provided by Gian Pietro di Sansebastiano (University of Salento, Lecce, Italy), who at short notice described their work on the syntaxin 5 proteins in *A. thaliana*. One of the puzzles in vesicle fusion is the apparent over-abundance of SNARE proteins relative to their requirement for vesicle fusion. He has proposed a new class of SNARE, the iSNARE of interfering SNARE that can become non-fusogenic when over-expressed [72]. In *A. thaliana* protoplasts SYP51 and SYP52 typically function as t-SNARES, however when localized to the tonoplast these proteins take on inhibitory or non-fusogenic roles. Different roles for these two highly similar SNARE proteins were also presented, identifying SYP51 as essential for trafficking to the central vacuole [75].

### 5.4. RAB Proteins

Several talks focused on a family of small GTPases, known as RAB proteins, which are frequently described as “master regulators” of membrane trafficking. RABs are highly conserved molecular switches that help to specify a unique identity for membranes to which they are transiently attached. Activated, GTP-bound RABs interact with a wide variety of other proteins known as “effectors”. Although their structure and core mechanisms are highly conserved, RAB proteins have diversified extensively between different organisms. Over 60 RAB genes have been identified in humans and their roles in regulating intracellular traffic throughout the endomembrane system is well known. However, less well established roles for RAB proteins include signaling, cell cycle control and cellular migration.

The group of Cinzia Progida (University of Oslo, Oslo, Norway) used human cells to develop cell migration and proliferation assays that could be used as the basis for an RNAi screen for RAB proteins. The RAB7 group is known to regulate transport between the late endosomes and the Golgi apparatus, and previous work by the Progida group had identified an additional role for RAB7b as a coordinator of cytoskeleton organization through direct interactions with myosin II and by activating RhoA and therefore actin remodeling [76]. As remodeling of the actin cytoskeleton is essential in cell migration, the work by Borg et al. [76] provides a strong foundation for deciphering the role of some RAB proteins in cell motility. Progida presented the results of the motility screens and control experiments for reduced cell proliferation. Their recent results demonstrating that one of the candidates, RAB7a, does regulate cell migration has just been published [77]. Like RAB7b, RAB7a also regulates the actin cytoskeleton, but through interactions with RAC1 and vimentin.

An alternative way to gain insight into the function of proteins is to physically isolate them and identify interacting partners. One classical method for establishing interactions is to use co-immunoprecipitation, where one protein is isolated by affinity purification using antibodies raised to either an epitope on the bait protein itself or—more commonly—to an established fusion protein such as GFP. Alex Jones (University of Warwick, Coventry, UK) presented data from a large proteomics dataset that used fluorescent proteins fused to seven target proteins and gentle protein extraction conditions to isolate not just direct protein interactions but large sections of the endomembrane system to which they are attached [78]. Heard et al. focused on four RAB baits, RABD2a/ARA5, RABF2b/ARA7, RABF1/ARA6, and RABG3f, as markers for combinations of the Golgi apparatus, trans Golgi network, early endosomes, secretory vesicles, late endosomes, multivesicular bodies, and the tonoplast. In addition, to compare these sub-proteomes with each other and other published proteomic datasets, she presented a few highlights of using interaction datasets such as STRING to further interrogate the data.

In plants, cell wall material and enzymes, such as xyloglucan, are cargos of RAB-regulated routes. A chemical genomic approach to study xyloglucan metabolism and trafficking in the model plant *A. thaliana* was then presented by Grégory Mouille (Institut Jean-Pierre Bourgin, INRA, Versailles, France). Well-defined polymeric xyloglucan structures are present in the plant cell wall. While core xyloglucan biosynthesis is located in the Golgi key maturation steps occur in the apoplast. Several cell wall residing enzymes that carry out the trimming are known and mutants are available. Grégory Mouille used sophisticated mass spectrometric technologies combined with chemical genomics (5000 compounds tested) to characterize the cell wall structure and phenotype of these mutants. This approach reveals uncharacterized subcellular compartments involved in the dynamic and polarized trimming of the cell wall polysaccharide [79,80].

### 5.5. AP-2 Adaptor Complex

George Diallinas (National and Kapodistrian University of Athens, Athens, Greece) focused on the unexpected function of the AP-2 adaptor complex outside of clathrin dependent endocytosis in hyphal tip growth of *Aspergillus*. In higher fungi, the AP-2 complex appears to have lost the clathrin binding domain (in β2 subunit). Nevertheless, it does interact with endocytosis markers, lipid flippases, sphingolipid and sterols biosynthetic machineries and contributes to proper apical membrane lipid composition. The question arisen during the meeting was if this imply a possibility of similar features in some other adaptor complexes.

### 5.6. Invariant Chain

To mount adaptive immune responses requires the cell surface expression of Major Histocompatibility Class II molecules loaded with antigenic peptide. Oddmund Bakke (University of Oslo, Oslo, Norway) reported on his previous studies focused on Major Histocompatibility Class II—associated Invariant chain (also known as CD74), which serves as a chaperone for Major Histocompatibility Class II molecules and mediates trafficking to the endosomal pathway in humans [81]. In fact, Invariant chain contains sorting signals within its cytoplasmic tail, which mediate its trafficking from PM to proteolytic endosomal compartments. He described also how the invariant chain can prolong the half-life of Major Histocompatibility Class II through its action on the endocytic pathway, suggesting that this alternative endocytic pathway induced by Invariant chain would serve to enhance the rate, quantity and diversity of Major Histocompatibility Class II antigen presentation.

This section is a miniature showcase of the diversity of topics that UPMT meeting attendees work on. With such diversity in proteins of the endomembrane system and organisms presented in this section, it can be hard to find a point of commonality. However, the molecular mechanisms of secretion and membrane trafficking, conventional or not, provided a thread of continuity between the subjects. The same concept can be applied to the next Section 6.

## 6. Unusual or Unexplored Intra- and Intercellular Pathways and Organelle Biogenesis

Intra- and intercellular communication is a fundamental mechanism for the coordination of organs during development and growth, stress adaptation, immunity and host-pathogen interactions in different biological kingdoms. Traffic and exchange of proteins, hormonal peptides and nucleic acids among cells play an essential role. Section 6 is characterized by talks in several research fields, showing how RNA/peptides as molecular signals regulate different processes in relation to trafficking mechanisms, including plant sexual reproduction or organelle function and dynamics.

### 6.1. Plant Sexuality

**Figure 5:**
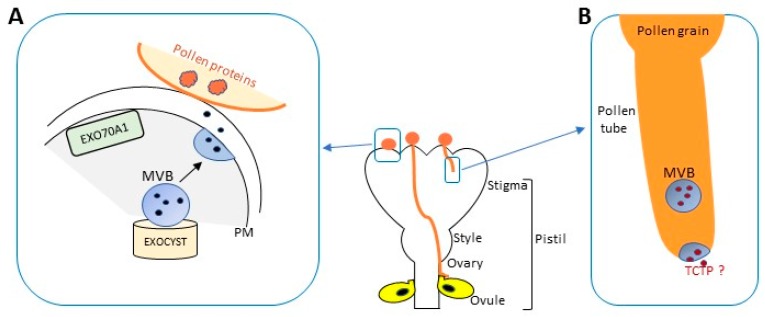
UPS seems to be involved in regulation of plant sexual reproduction. (**A**) Simplified scheme, modified from [83], of compatible pollen response pathway in Brassica. Exocyst dependent secretion was shown to be important for the plant stigma receptivity. The octameric exocyst complex at the stigma–pollen grain contact sites mediates secretion of factors stimulating pollen hydration and germination, by non-conventional secretion of exosomes via multivesicular bodies (MVB) fusion with the PM. EXO70A1 is a subunit of the exocyst complex which functions as a tethering complex to mediate polar secretion. Specific pollen proteins are proposed to mediate the recognition of compatible pollen, but the corresponding receptor is still unknown; (**B**) Pollen tube-pistil signaling in *A. thaliana*. A hypothesis is that the translationally controlled tumor protein (TCTP), a leaderless secretory protein, probably released via exosomes, is involved in male–female recognition, indicating a putative role of UPS in pollen tube guidance towards the ovules for fertilization.

Daphne Goring (University of Toronto, Toronto, Canada) focused on the exocyst, exosomes and autophagy in the regulation of plant sexual reproduction (Figure 5). Normal exocyst dependent secretion was shown to be important for the plant stigma receptivity. Reported observations indicate that the exocyst at the stigma–pollen grain contact sites mediates secretion of factors stimulating pollen hydration and germination [82,83]. In both compatible as well as incompatible pollinations within the family Brassicaceae, also a non-conventional secretion of exosomes via multivesicular bodies fusion with the PM is implied. In the incompatible pollinations, one of the exocyst subunits EXO70A1 is degraded and the autophagy pathway is activated as a part of active self-pollen recognition and rejection. Furthermore, Said Hafidh (Institute of Experimental Botany ASCR, Prague, Czech Republic) showed pollen tube-pistil signaling during sexual reproduction in *A. thaliana* plants [84], suggesting that leaderless secretory proteins, probably released via exosomes, dominate key processes in male–female recognition. His data, regarding the exosomes mediated transport of translationally controlled tumor protein in *A. thaliana*, indicate a putative role of UPS in pollen tube guidance towards the ovules for fertilization.

### 6.2. EXPO

Liwen Jiang (Chinese University of Hong Kong, Hong Kong, China) debated the crosstalk existing between UPS and autophagy, describing multiple transport pathways and showing how distinct mechanisms regulate the conventional secretory pathway and UPS in plant cells [85]. Plant cells are particularly interesting for these types of studies because they display a large number of compartments that can support research on UPS mechanisms and multivesicular bodies formation [86], even if our understanding of UPS mechanisms in plants is still very limited. An example of vesicular UPS mediated by a plant-specific double-membrane-bound compartment is the contentious exocyst-positive organelle (EXPO), which biogenesis seems to be mediated by a UPS pathway and crosstalk with the autophagic pathway [4]. The EXPO pathway was disputed after the talk and in the poster session and offers a good example of unconventional traffic to be fully elucidated.

### 6.3. Mitochondrion

Cristiano Simone (University of Bari, Bari, Italy) discussed the unconventional transport of FoxO3A, a transcription factor working in normal conditions on genomic DNA in the nucleus, to mitochondria during nutrient deprivation in a process dependent of MEK/ERK and the AMPK pathways. These kinases provide the final signature by phosphorylating serines 12 and 30 on FoxO3A N-terminal domain, which is required for the recruitment to the mitochondrial membrane. After that, FoxO3A is imported and processed by the mitochondrial processing peptidase system. Inside mitochondria, FoxO3A activates the transcription of the mitochondrial genome, thus leading to increased O_2_ consumption.

### 6.4. Chloroplast

Jürgen Soll and his coworkers (Ludwig-Maximilians-Universität München, Munich, Germany) use biochemical and electron microscopic techniques to uncover transport and signal transduction processes in green plants [87]. One focus is on chloroplasts and its integration into the cell. The chloroplast contains an extensive thylakoid membrane system that is critical for photosynthesis. During chloroplast differentiation from non-green proplastids the complex internal membrane system is formed. This process involves vesicle formation and membrane invagination [88]. In his presentation, Soll summarized the current knowledge on chloroplast vesicle transport and thylakoid biogenesis. He stressed that despite of extensive research in this area not much is known about this unusual membrane system and while bioinformatics predict their presence no single protein factor involved in the formation has been identified yet.

### 6.5. RNA as Molecular Signals in Plant Biology

Daniel Garcia Cabanillas (INRS Institut Armand-Frappier, Laval, QC, Canada) covered the replication of the Turnip mosaic virus, a positive-sense (+) RNA plant virus, which induces substantial endomembrane system remodeling during infection. The virus, indeed, is known to induce ER-derived vesicles, commonly known as “viral replication factories”, which house viral RNA as well as viral and host proteins required for its replication. These vesicles are also involved in the intercellular trafficking of the Turnip mosaic virus and are released at the ER in a COPII-dependent transport. The viral membrane associated protein 6K2 has an essential role in vesicle formation [89]. The work of Cabanillas was focused on this protein, identifying a transmembrane stretch of five amino acids, GxxxG, responsible for the 6K2 protein bypassing the Golgi apparatus. The substitution of the glycine residues with valine resulted in a delocalization of the protein in the Golgi apparatus and PM and prevented normal replication vesicle production. Cabanillas suggested a “tug of war” between conventional and unconventional trafficking pathways during virus replication, which was confirmed by an increase of virus cell-to-cell movement in dominant negative mutants of Golgi SNAREs where the ER-Golgi traffic is disrupted. The presentation by Julia Kehr (Hamburg University, Hamburg, Germany) can be seen as a corollary to this previous report. She presented an overview of the role of RNA as a molecular signal in plant biology [55,90], discussing results on the intercellular transport of the RNA and its role in plant development and defense reactions against pathogens. She also showed different experiments in which several RNAs have been identified in the phloem, suggesting that mobile RNAs can act as long-distance signals in higher plants and that siRNAs and miRNAs can be involved in plant-virus interactions during the infection process.

### 6.6. Other Examples of Unusual or Unexplored Intra- and Intercellular Pathways

Valeria Crippa from the Angelo Poletti’s lab (CEND University of Milano, Milano, Italy) presented results devoted to the function of small heat shock protein chaperon B8 in autophagic disposal of misfolded proteins implied in motor neuron diseases. The B8 protein, upregulated in motor neuron diseases patients, was shown to increase an autophagic/lysosomal degradation of misfolded proteins in a complex with co-chaperons (including E3 ligase CHIP) in cellular models and a clear protective function in *Drosophila* model was also observed [91]. Moving to autophagy induced by nutrient starvation, Xiaoqiang Yao (Chinese University of Hong Kong, Hong Kong, China) reported on a new potassium channel crucial for this kind of autophagy in animal cells. The K^+^ channel TM9SF4 is especially abundant in kidneys and its knockdown inhibits autophagy normally activated via mTOR signaling pathway.

Andrea Pompa (Institute of Biosciences and Bioresources, CNR, Perugia, Italy) presented unpublished data on the maturation and unconventional trafficking of the CLV3 protein in transgenic tobacco plants. The CLV3 protein is the precursor of a small 12 amino acid active peptide recognized at the cell surface by CLV1/2 receptor complexes [92] and cooperates in a signaling mechanism that limits excessive proliferation of pluripotent cells in plant apical meristems [93]. Despite the presence of a signal peptide, CLV3 does not follow the conventional secretory pathway, but is retro-translocated from ER to the cytosol. In this process, the proteasome machinery seems to have a role in the production of the active CLV3 peptide. Furthermore, he postulated that the active ligand, once released into the cytosol, reaches the apoplast by an unconventional secretion process yet to be discovered.

Muhammad Akbar Abdul Ghaffar (Ohio State University, Columbus, OH, USA) described another process of intracellular trafficking. He applied transmission electron microscopy to study rubber ontogeny in the alternative rubber crop *T. kok-saghyz*. Natural rubber is synthetized and compartmentalized in particles of some plants and fungi, but the ontogeny and development of these particles is not well understood [94]. In contrast to the current main rubber producer *Hevea brasiliensis* or its alternative *Parthenium argentatum* growing in arid regions, the plant *Taraxacum* would be of interest for rubber production in temperate areas like parts of the US or Europe. Muhammad Ghaffar presented first insights into rubber ontogeny in secretory laticifer cells of *Taraxacum*. He observed rubber being initially formed in the ER-Golgi membrane system. Rubber particles were also found in vesicles located in the cytoplasm and in plastids of laticifer cells. The findings contribute to understanding rubber ontogeny and hence to establishing *T. kok-saghyz* as a novel rubber producer.

## 7. Conclusions

The first “Unconventional Protein and Membrane Traffic” meeting was something of a gamble with a happy conclusion. The UPS topic is intrinsically very diverse and heterogeneous because it involves many pathways, cargoes, cell types, and triggering conditions. Moreover, the organizers had not been sure that bringing together scientists working on different model systems, from primary human monocytes to yeast cells, or from the plant *A. thaliana* to the fungus *U. maydis*, was a good idea because the audience might struggle to follow the talks if the speaker’s research background is too distant from their own research field. Occasionally this might have been the case, but in general the meeting was very successful and on the last day, during an open final discussion, there was unanimity among the participants that the second UPMT meeting should be held in 2018.
